# Supplementary figures and images for: Severe outcomes of COVID-19 among adults with increased risk conditions: A population-based observational study
Source: PLoS One. 2025 Feb 11;20(2):e0316529. doi: 10.1371/journal.pone.0316529 (PMC11813104; doi:10.1371/journal.pone.0316529)

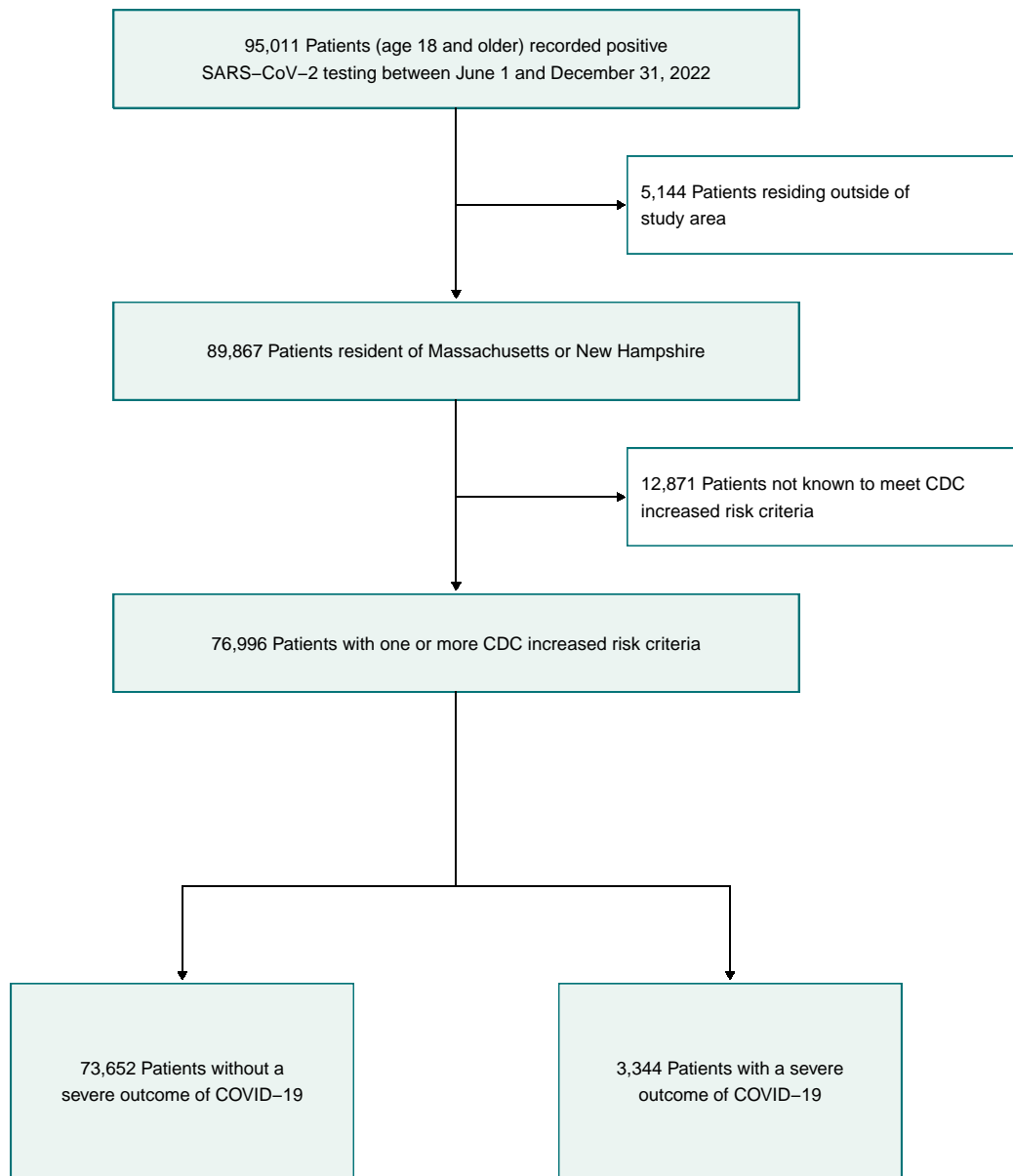

Supplement: S1 Fig — (PDF) [file pone.0316529.s001.pdf]
