# Supplementary material for: Severe outcomes of COVID-19 among adults with increased risk conditions: A population-based observational study
Source: PLoS One. 2025 Feb 11;20(2):e0316529. doi: 10.1371/journal.pone.0316529 (PMC11813104; doi:10.1371/journal.pone.0316529)
Supplement: S1 Table — (DOCX) [file pone.0316529.s002.docx]

| **Supplemental Table 1. Odds ratios of preceding outpatient diagnosis of COVID-19 among patients with severe outcomes^a^ by selected characteristics— Mass General Brigham, June to December 2022** | | | |
| --- | --- | --- | --- |
| **Characteristic** | **Odds Ratio**^b^ | **95% CI** | **p-value** |
| Comorbidity score |  |  | 0.2 |
| MASS 3 or less | — | — |  |
| MASS 4 and 5 | 0.97 | 0.76, 1.23 |  |
| MASS 6 or greater | 0.92 | 0.74, 1.13 |  |
| Severe immunocompromise | 1.17 | 0.92, 1.49 |  |
| Age |  |  | <0.001 |
| 18 to 49 | — | — |  |
| 50 to 64 | 0.81 | 0.64, 1.03 |  |
| 65 to 79 | 0.68 | 0.54, 0.86 |  |
| 80 and older | 0.62 | 0.49, 0.79 |  |
| Vaccination status |  |  | 0.002 |
| Unvaccinated or fewer than 3 doses | — | — |  |
| Vaccinated, last dose or COVID-19 ≥ 8 months prior | 1.09 | 0.90, 1.33 |  |
| Vaccinated, last dose or COVID-19 < 8 months prior | 1.44 | 1.16, 1.79 |  |
| Race and Ethnicity |  |  | 0.3 |
| Black, Latinx, and other | — | — |  |
| White and Asian | 1.09 | 0.91, 1.31 |  |
| Neighborhood disadvantage (ADI) |  |  | 0.010 |
| 1st to 74th percentile | — | — |  |
| 75th or higher percentile | 1.29 | 1.06, 1.56 |  |
| Outpatient COVID-19 treatment |  |  | <0.001 |
| No treatment | — | — |  |
| Received treatment | 7.22 | 6.03, 8.66 |  |
| Abbreviations: ADI, Area Deprivation Index; CI, confidence interval ^a^ Recorded positive COVID-19 test result two or more calendar days prior to COVID hospitalization or death. ^b^ Adjusted odds ratios from multivariable logistic model of apriori-selected characteristics are shown. | | | |
